# Effect of ACE, ACE2 and CYP11B2 gene polymorphisms and noise on essential hypertension among steelworkers in China: a case–control study

**DOI:** 10.1186/s12920-022-01177-0

**Published:** 2022-02-08

**Authors:** Xiaohong Zhang, Ying Wang, Yao Zheng, Juxiang Yuan, Junwang Tong, Jingya Xu, Qinglin Li, Peishuai Li, Shoufang Jiang, Zhaoyang Wang, Feng Chai, Xiangwen Li

**Affiliations:** 1 School of Public Health, North China University of Science and Technology, No. 21 Bohai Avenue, Caofeidian Area, Tangshan, 063210 Hebei Province People’s Republic of China; 2 Department of Endocrinology, North China University of Science and Technology Affiliated Hospital, Tangshan, People’s Republic of China; 3 Department of Public Health, North China University of Science and Technology Affiliated Hospital, Tangshan, People’s Republic of China; 4 Tangshan Hongci Hospital, Tangshan, People’s Republic of China

**Keywords:** Essential hypertension, Noise, Gene polymorphism, Interaction, GMDR

## Abstract

**Background:**

Previous studies on the relationship between ACE I/D, ACE2 G8790A and CYP11B2-344T/C gene polymorphisms and essential hypertension (EH) were inconsistent. Moreover, few studies have reported the combined effect of these gene polymorphisms and noise exposure on EH. The purpose of this study was to explore the combined and separate effects of ACE I/D, ACE2 G8790A and CYP11B2-344T/C gene polymorphisms and noise on EH among steelworkers.

**Methods:**

A case–control study was conducted on 725 male workers between March 2014 and July 2014 in the Tangsteel Company, China. The noise exposure of the workers were measured. Logistic regression and crossover analysis were used to analyse the effects of the interactions on the EH among steelworkers. GMDR was used to determine the best combination model of gene–noise interactions.

**Results:**

Multivariate logistic regression showed that noise exposure increased the odds of EH, and the OR is 1.52 (95% CI 1.04–2.22). The risk of having EH for ACE I/D DD genotype carriers was 1.99 times that for II genotype carriers (95% CI 1.14–3.51). There was a negative additive interaction between ACE2 G8790A and CYP11B2-344T/C on EH (U3 =  − 2.221, *P* = 0.026, and S = 0.128) and a positive multiplicative interaction between ACE I/D and CYP11B2-344T/C on essential hypertension (*P* = 0.041). In addition, there was no significant gene–noise interaction model through the GMDR method after adjusting the confounders.

**Conclusions:**

The ACE DD genotype may make men susceptible to EH. Simultaneously carrying the DD genotype of ACE I/D and the TC genotype of CYP11B2-344T/C increased the risk of EH.

**Supplementary Information:**

The online version contains supplementary material available at 10.1186/s12920-022-01177-0.

## Background

Essential hypertension (EH) is one of the most common cardiovascular diseases and is affected by a variety of genetic and environmental factors [1]. In recent decades, the prevalence of hypertension among Chinese adults has risen sharply, making it a public health problem that cannot be ignored [2]. Noise is common in workplaces in the steel industry. Previous studies have shown that noise can increase plasma renin, angiotensin II and aldosterone concentrations in the body, causing excessive activation of the renin–angiotensin–aldosterone system (RAAS) [3], thereby promoting the occurrence and development of hypertension [4]. The RAAS plays an important role in regulating human blood pressure. RAAS genes are the most likely susceptibility genes for essential hypertension [5, 6], such as angiotensin–converting enzyme, angiotensin–converting enzyme 2, and aldosterone synthase. Studies have shown that the ACE I/D polymorphism [7], the ACE2 G8790A polymorphism [8], and the CYP11B2-344T/C polymorphism [9] are related to the occurrence of hypertension. However, some studies have yielded inconsistent results [10, 11], and few studies have reported the interaction of ACE polymorphism, ACE2 polymorphism, CYP11B2 polymorphism with noise exposure on hypertension. In this study, a case–control study was carried out among steelworkers, in whom the interactions between noise exposure and the ACE I/D, ACE2 G8790A, and CYP11B2-344T/C polymorphisms were analysed. The effects of gene–gene interactions on EH were also analysed.

## Methods

### Study population

A case–control study was conducted in Tangsteel Company in Tangshan, China. In the study, the selected subjects were male workers who participated in the occupational health examination of the steel company between March 2014 and July 2014.

EH was diagnosed according to the preventive report of the seventh US Joint National Committee on Detection, Evaluation and Treatment of Hypertension (JNC 7) as the diagnostic criteria [12], combined with the workers' medical history and the history of taking hypertension medication. Inclusion criteria for the case group: (1) the patient was diagnosed with EH according to the diagnostic criteria; (2) was a Han male worker who was 18 years old or older; and (3) had worked at the Tangsteel Company for at least 1 year. Exclusion criteria: (1) the participant had a history of liver or kidney disease, diabetes, secondary hypertension, or other cardiovascular diseases; (2) was exposed to high temperatures; or (3) had been employed in a different line of work before. Those in the control group were diagnosed as non-hypertensive according to the same diagnostic criteria, and the other inclusion and exclusion criteria were the same as those in the case group. A total of 725 subjects were eventually included; among them, 224 were in the case group and 501 were in the control group.

1 $$\begin{array}{*{20}c} {n = \frac{{\left[ {z_{ \propto } \sqrt {2\overline{p}(1 - \overline{p})} + z_{\beta } \sqrt {p_{1} (1 - p_{1} ) + p_{0} (1 - p_{0} )} } \right]^{2} }}{{(p_{1} - p_{0} )^{2} }}} \\ \end{array}$$

2 $$\begin{array}{*{20}c} {\overline{p} = \frac{{(p_{1} + p_{0} )}}{2}} \\ \end{array}$$

3 $$\begin{array}{*{20}c} {p_{1} = \frac{{(OR \times p_{0} )}}{{(1 - p_{0} + OR \times p_{0} )}}} \\ \end{array}$$

*p*_*0*_: The exposure rate of control group;

*p*_0_: The exposure rate of case group.

In this study, we initially screened the data according to the National Center for Biotechnology Information (NCBI) Single Nucleotide Polymorphism (SNP) database (http://www.ncbi.nlm.nih.gov/SNP) and the minimum allele frequency (MAF) of the selected SNP locus was 0.216. We calculated the sample size and followed by the conditions: α = 0.05, β = 0.10, expected OR = 2.0, and the calculated sample size is 218 in the case group and control group, respectively. The present sample size is enough.

This study was approved by the Ethics Committee of North China University of Science and Technology, and all the subjects voluntarily signed an informed consent form.

### Questionnaire and assessment of covariates

In this study, uniformly trained personnel surveyed all the research subjects in face-to-face interviews that followed a uniform questionnaire. The contents of the questionnaire mainly included: (1) general demographic information, including age, sex, height, weight, marital status, education level and family income; (2) occupation history, including working-age and shift; (3) lifestyle and eating habits, including smoking (never smoked vs. had quit or still smoked), drinking (never drank vs. had quit or still drank), fried food, meat, whole grains, fruit, vegetable intake and physical exercise (< 3 times/week is less, ≥ 3 times/week is more); (4) psychological stress, including work, family, economic and emotional stress (sometimes with or without being light, periodic or always being heavy); and (5) personal disease history and family history of hypertension.

The subjects of the study were examined by a group of uniformly trained researchers according to the standard of the instrument, including height, weight, blood pressure, heart rate and electrocardiogram. Then the body mass index (BMI) was calculated as weight (kg) divided by the height squared (m^2^).

Venous blood of the workers was collected from the anterior elbow, anticoagulated with EDTA, and stored in a refrigerator at − 80 °C for DNA extraction and biochemical analysis. The total cholesterol (TC) and triglyceride (TG) in the serum were measured.

### Determination of noise and high-temperature exposure

Noise exposure of the workplace was measured using a sound analyser (TES-1350A; TES Electronic Corp, Taiwan). Time-weighted average (TWA) noise defined a worker’s occupational noise exposure time of 8 h per day or 40 h of work per week based on the time spent in each location and the average noise level. In our study, the 40-h TWA level was calculated from the worker’s staying time in the workplace and the shift situation. If the noise exposure was greater than or equal to 80 dB (A), the subjects were judged to be noise-exposed. According to Zhao et al. [13], cumulative noise exposure (CNE) was calculated as follows:

4 $$\begin{array}{*{20}c} {CNE = 10 \times \log (\sum 10^{{0.1 \times {\text{L(A)eq}}}} \times {\text{the}}\;{\text{years}}\;{\text{of}}\;{\text{noise}}\;{\text{exposure}})} \\ \end{array}$$

The temperature of the workplace was measured with a wet bulb global temperature (WBGT) analyser (QT-36; Quest Technologies Corp, America) in July and August 2014, the two hottest months of the year. The temperature of different workspaces was detected, from which each worker's WBGT value was calculated. In this study, if the WBGT was greater than or equal to 25 °C and the subjects were exposed to productive heat sources, they were judged to be high-temperature workers.

### ACE I/D, ACE2 G8790A, CYP11B2-344T/C gene polymorphism detection

The DNA of the blood sample was extracted by using a whole-blood rapid genomic DNA extraction kit (Beijing Aid Lab Biotechnology Co, Ltd.). Gene polymorphisms were typed by restriction fragment length polymorphism (RFLP) techniques and polymerase chain reaction (PCR). The total volume of the amplification reaction was 25 μl, including 12.5 μl PCR Taq Master Mix, 1.0 μl upstream primer, 1.0 μl downstream primer, 1.0 μl DNA template, and 9.5 μl deionized water.

**Fig. 1:**
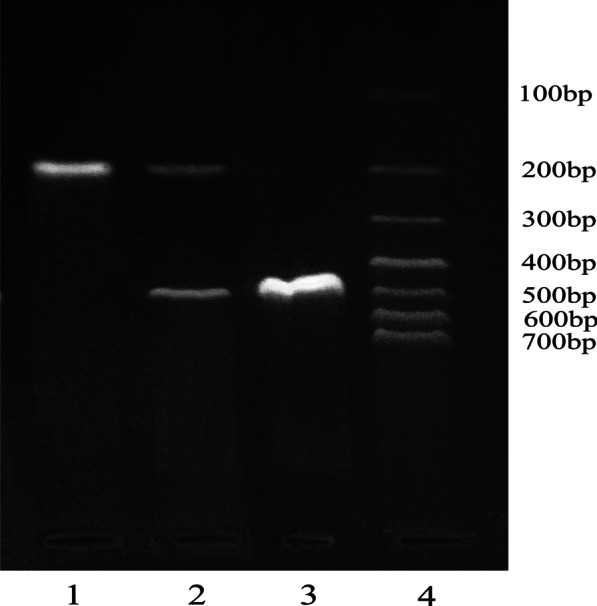
Agarose gel (2%) electrophoresis of ACE I/D. (1) DD(190 bp); (2) ID(190 bp + 490 bp); (3) II(490 bp); 4: DNA marker

**Fig. 2:**
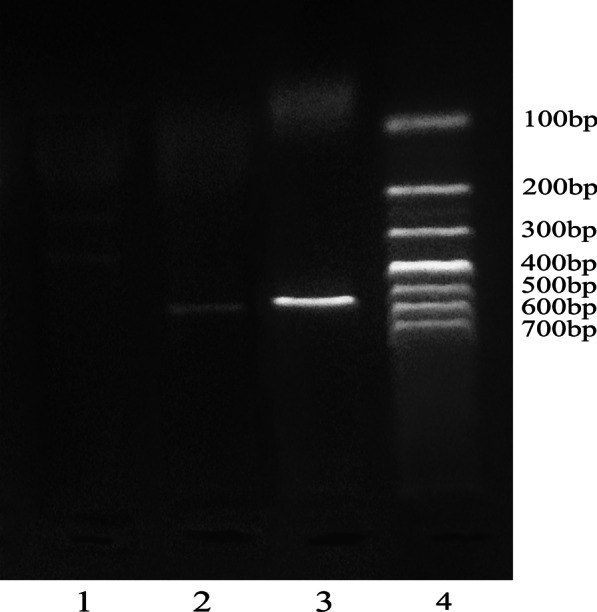
Agarose gel (2%) electrophoresis of ACE2 G8790A. (1) AA(281 bp + 185 bp); (2) GG(466 bp); (3) PCR products (466 bp); (4) DNA marker

**Fig. 3:**
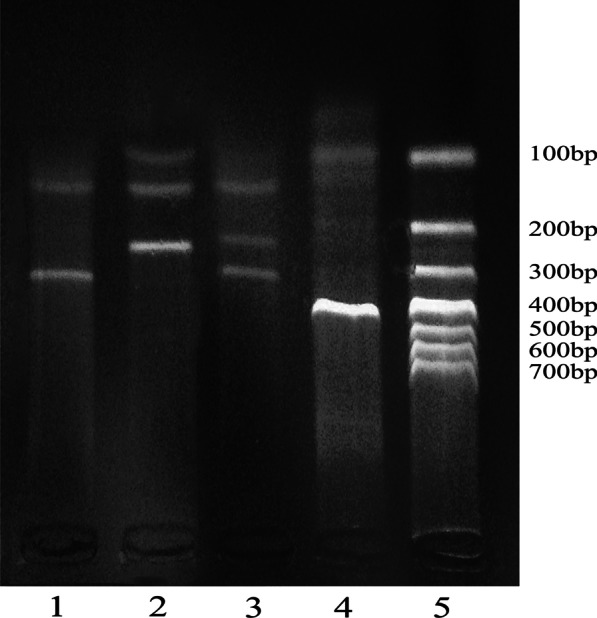
Agarose gel (2%) electrophoresis of CYP11B2–344T/C. (1) TT(116 bp + 283 bp); (2) CC(71 bp + 116 bp + 212); (3) TC(71 bp + 116 bp + 212 bp + 283 bp); (4) PCR product; (5) DNA marker

Primers for PCR were designed using the Primer 5.0 software package (Beijing Ruiboxingke Biotechnology Co, Ltd.). The primer sequences and PCR conditions of each gene locus are shown in Additional file 1: Table S1 and Table S2 in the supplement file, and each PCR product needed to be digested with restriction enzymes (Additional file 1: Table S2). Because ACE I/D is an insertion/deletion site, the genotyping results could be seen by directly observing the amplification products of PCR (Fig. 1). The PCR products of ACE2 G8790A and CYP11B2-344T/C were reacted with the AluI endonuclease (NEB Corp, American) and the HaeIII endonuclease (NEB Corp, American) in a water bath at 37 °C for 3 h, electrophoresed on a 2.0% agarose gel and stained with ethidium bromide. Genetic polymorphisms were detected with an ultraviolet analyser, as shown in Fig. 2 and Fig. 3.

### Statistical analyses

For the comparison of continuous baseline characteristics, Student’s t test or Wilcoxon’s rank sum test was used. For categorical variables, the chi-square test was used for comparison. The Hardy–Weinberg equilibrium (HWE) test and comparison of the distribution of alleles and genotypes between the case and control groups were performed with the chi-squared test. The effects of the interaction between gene and gene and between gene and noise based on multiplicative and additive models on EH were investigated by logistic regression and the crossover analytical method proposed by Rothman et al. [14]. Generalized multifactor dimensionality reduction (GMDR, 0.9) was used to determine the best-combined model of the interaction between genes and noise. Analyses were conducted with SPSS 23.0 (SPSS Inc., Chicago, IL, USA) with a two-sided significance threshold of *P* < 0.05.

## Results

### Comparison between workers of the EH group and control group

Comparing the two groups, the percentage of workers exposed to noise in the EH group (42.0%) was significantly higher than that in the control group (31.6%) (*P* < 0.05). The percentage of workers with a family history of hypertension in the EH group (43.4%) was significantly higher than that in the control group (29.2%) (*P* < 0.01). The percentage of obesity in the EH group (35.3%) was higher than that in the control group (12.0%), and the distribution of BMI between the two groups was significantly different (*P* < 0.05). Age, length of service, TC and TG in the EH group were significantly higher than those in the control group (*P* < 0.05). The percentage of the EH group (18.4%) with a university education and above was significantly lower than that of the control group (33.2%) (*P* < 0.01). The percentages of workers who regularly did physical exercise and regularly ate vegetables in the EH group were lower than those in the control group (*P* < 0.05). The other factors, including marital status, shift work, smoking and drinking, were not significantly different, as shown in Table 1.

**Table 1:** Comparison of the baseline data between workers of EH group and control group

Factors	Case, n (%)	EH group (*n* = 224)	Control group (*n* = 501)	*t*/*χ*^2^/*U*	*P*
Noise, n (%)				7.422	0.006
No	473	130 (58.0)	343 (68.4)		
Yes	252	94 (42.0)	158 (31.6)		
BMI (kg/m^2^), n (%)				72.117	< 0.001
< 24	265	42 (18.7)	223 (44.5)		
24–27	321	103 (46.0)	218 (43.5)		
≥ 28	139	79 (35.3)	60 (12.0)		
Marriage, n (%)				3.458	0.063
No	67	14 (6.2)	53 (10.5)		
Yes	658	210 (93.8)	448 (89.5)		
Education, n (%)				16.69	< 0.001
Below university	518	183 (81.6)	335 (66.8)		
University and above	207	41 (18.4)	166 (33.2)		
Income, n (%)				1.126	0.289
< 3000	538	172 (76.7)	366 (73.0)		
≥ 3000	187	52 (23.3)	135 (27.0)		
Current shift status, n (%)				0.311	0.577
No	224	66 (29.4)	158 (31.5)		
Yes	501	158 (70.6)	343 (68.5)		
Family history of hypertension, n (%)				13.932	< 0.001
No	482	127 (56.6)	355 (70.8)		
Yes	243	97 (43.4)	146 (29.2)		
Smoking				0.952	0.329
No	314	91 (40.6)	223 (44.5)		
Yes	411	133 (59.4)	278 (55.5)		
Drinking				2.494	0.114
No	582	172 (76.7)	410 (81.8)		
Yes	143	52 (23.3)	91 (18.2)		
Eat fried food				2.364	0.124
< 3 times/week	644	205 (91.5)	439 (87.6)		
≥ 3 times/week	81	19 (8.5)	62 (12.4)		
Eat meat				1.667	0.197
< 3 times/week	421	138 (61.7)	283 (56.4)		
≥ 3 times/week	304	86 (38.3)	218 (43.6)		
Eat whole grains				0.110	0.741
< 3 times/week	591	181 (80.8)	410 (81.8)		
≥ 3 times/week	134	43 (19.2)	91 (18.2)		
Eat vegetable				5.094	0.024
< 3 times/week	139	54 (24.2)	85 (16.9)		
≥ 3 times/week	586	170 (75.8)	416 (83.1)		
Eat fruit				0.785	0.376
< 3 times/week	274	90 (40.1)	184 (36.7)		
≥ 3 times/week	451	134 (59.9)	317 (63.3)		
Physical exercise				5.019	0.025
< 3 times/week	166	63 (28.2)	103 (20.6)		
≥ 3 times/week	559	161 (71.8)	398 (79.4)		
Work pressure				0.005	0.945
Smaller	448	138 (61.6)	310 (61.8)		
Larger	277	86 (38.4)	191 (38.2)		
Family pressure				0.138	0.710
Smaller	626	195 (87.0)	431 (86.0)		
Larger	99	29 (13.0)	70 (14.0)		
Economic pressure				0.620	0.431
Smaller	533	169 (75.4)	364 (72.6)		
Larger	192	55 (24.6)	137 (27.4)		
Emotional stress				0.545	0.460
Smaller	693	216 (96.4)	477 (95.2)		
Larger	32	8 (3.6)	24 (4.8)		
Age (years)		40.86 ± 8.15	36.98 ± 8.08	41,213.500	< 0.001
Length of service		20.58 ± 9.45	16.35 ± 9.17	41,478.000	< 0.001
TG (mmol/L)		1.76 (1.34, 2.26)	1.36 (0.98, 2.06)	41,926.500	< 0.001
TC (mmol/L)		4.90 (4.30, 5.60)	4.70 (4.10, 5.30)	48,575.000	0.004

BMI, body mass index; EH, essential hypertension; TC, total cholesterol; TG, triglyceride

The genotype distributions at ACE I/D, ACE2 G8790A, and CYP11B2-344T/C in the control group all complied with the Hardy–Weinberg equilibrium law. Compared with the control group, the EH group had a significantly different ACE I/D genotype distribution (*P* < 0.05). The percentage of the D allele in the EH group was significantly higher than that in the control group (*P* < 0.05). Because ACE2 G8790A is on the X chromosome, men only have the GG genotype or AA genotype. There was no statistically significant difference in the genotype distribution of ACE2 G8790A in the EH group. The percentage of the A allele in the EH group was significantly higher than that in the control group (*P* < 0.05). There was no statistically significant difference in the genotype percentage or allele percentage of CYP11B2-344T/C between groups see Additional file 1: Table S3.

### Multivariate logistic regression analysis of the relationship between environmental factors, gene polymorphisms and EH

The statistically significant indicators in the univariate logistic regression analysis were input as independent variables and EH was taken as the dependent variable to perform multiple logistic regression analysis to eliminate the influence of confounding factors.

The risk of having EH for ACE I/D DD genotype carriers was 1.99 times that for II genotype carriers (95% CI 1.14–3.51), which suggested that the DD genotype may be a susceptibility genotype for EH. The genetic polymorphisms CYP11B2-344T/C and ACE2 G8790A showed no association with essential hypertension (Table 2).

**Table 2:** Multiple logistic regression analysis of the relationship between noise exposure, gene polymorphisms and EH

Factors	Genotype	*OR* (95% CI)	*OR*^a^ (95% CI)
ACE			
	II	1.00	1.00
	ID	1.19 (0.85–1.68)	1.10 (0.74–1.63)
	DD	1.95 (1.19–3.20)	1.99 (1.14–3.51)
ACE2			
	GG	1.00	1.00
	AA	1.31 (0.95–1.79)	1.22 (0.85–1.76)
CYP11B2			
	TT	1.00	1.00
	TC	0.96 (0.69–1.34)	0.99 (0.68–1.45)
	CC	0.75 (0.41–1.37)	0.64 (0.32–1.28)
Noise			
	No	1.00	1.00
	Yes	1.57 (1.13–2.17)	1.52 (1.04–2.22)
BMI			
	< 24		1.00
	24–27	2.51 (1.67–3.76)	2.66 (1.72–4.13)
	≥ 28	6.99 (4.37–11.19)	7.98 (4.74–13.44)
Family history of hypertension			
	No		1.00
	Yes	1.86 (1.34–2.58)	1.87 (1.29–2.72)
Education			
	Below university		1.00
	University and above	0.45 (0.31–0.67)	0.57 (0.36–0.91)
Eat vegetable			
	< 3 times/week		1.00
	≥ 3times/week	0.64 (0.44–0.95)	0.57 (0.37–0.88)
Physical exercise			
	< 3 times/week		1.00
	≥ 3times/week	1.51 (1.05–2.17)	1.54 (1.00–2.36)
Age		1.06 (1.04–1.08)	1.08 (1.03–1.14)
Length of service		1.05 (1.03–1.07)	0.99 (0.94–1.03)
TG		1.22 (1.11–1.36)	1.20 (1.08–1.34)
TC		0.99 (0.98–1.01)	0.99 (0.95–1.03)

*OR*^a^: *adjusted* BMI, eduation, hypertension family history, eat vegetable, physical exercise, age, length of service, TG, TC

### Analysis of gene–gene interactions

From Table 3, the hypothesis testing of the interaction showed that ACE I/D and CYP11B2-344T/C showed no interaction in the additive model (U = 1.769, *P* = 0.076). ACE and ACE2 G8790A also did not have any interaction based on the additive model (U2 =  − 0.401, *P* = 0.688). ACE2 G8790A and CYP11B2-344T/C had an interaction based on the additive model (U3 =  − 2.221, *P* = 0.026). Among them, the synergy index S = 0.128, and there seemed to be a negative interaction between the two; the absolute value of the attribution proportion of the interaction (AP) was 0.856; the absolute value of the relative excess risk of interaction (RERI) was 0.979; and the interaction odds ratio (OR_INT_) = 0.485. EH was taken as the dependent variable, and each gene and its interaction terms were introduced into the logistic regression equation to analyse the influence of the interactions between ACE, ACE2 and CYP11B2 in the effect on EH based on the multiplicative model after adjustment for confounders such as BMI, family history of hypertension, TG, and TC.

**Table 3:** Analysis of the additive interactions between ACE, ACE2 and CYP11B2

Genetype	Genetype	Number of EH	Number of control	OR
ACE	CYP			
II	TT	53	114	1.00
II	TC + CC	33	113	0.628
ID + DD	TT	69	148	1.003
ID + DD	TC + CC	69	126	1.178
ACE	ACE2			
II	GG	37	121	1.00
II	AA	49	106	1.512
ID + DD	GG	72	156	1.509
ID + DD	AA	66	118	1.829
ACE2	CYP11B2			
GG	TT	58	164	1.00
GG	TC + CC	51	113	1.276
AA	TT	64	98	1.847
AA	TC + CC	51	126	1.144

As shown in Additional file 1: Table S4, ACE I/D and CYP11B2-344T/C showed an interaction based on the multiplicative model (*P* < 0.05); the risk of having EH for dual ACE I/D DD and CYP11B2-344T/C TC genotype carriers were 3.04 times that for dual ACE I/D II genotype and CYP11B2-344T/C TT genotype carriers (95% CI 1.25–7.39). The remaining interactions were not significant based on the multiplicative model in this study.

### Analysis of gene–noise interaction

From Table 4, The hypothesis testing of the interaction showed that none of the three genes showed an interaction with noise based on the additive model (ACE:U =  − 0.067, *P* = 0.946; ACE2:U =  − 0.289, *P* = 0.772; CYP:U =  − 0.896, *P* = 0.370).

**Table 4:** Analysis of additive interactions between ACE, ACE2, CYP11B2 and noise

Gene	Genetype	Noise	Number of EH	Number of control	OR
ACE	II	< 80 dB(A)	57	173	1.00
	II	≥ 80 dB(A)	29	54	1.630
	ID + DD	< 80 dB(A)	73	170	1.303
	ID + DD	≥ 80 dB(A)	65	104	1.897
ACE2	GG	< 80 dB(A)	68	204	1.00
	GG	≥ 80 dB(A)	41	73	1.685
	AA	< 80 dB(A)	62	139	1.338
	AA	≥ 80 dB(A)	53	85	1.871
CYP11B2	TT	< 80 dB(A)	69	183	1.00
	TT	≥ 80 dB(A)	53	79	1.779
	TT + CC	< 80 dB(A)	61	160	1.011
	TT + CC	≥ 80 dB(A)	41	79	1.376

Taking EH as the dependent variable, multiple-factor logistic regression analysis was used to analyse the influence of the interactions between ACE, ACE2, CYP11B2 and noise on EH based on the multiplicative model after adjustment for BMI, family history of hypertension, TG, TC and other confounders. None of the three genes showed an interaction with noise based on the multiplicative model see Additional file 1: Table S5.

### GMDR analysis of gene–noise interactions

The results showed that the four-factor model composed of ACE, ACE2, CYP genes and noise was the best model. The cross-validation consistency was 10/10, the test balance accuracy was 0.5084, and *P* = 0.3770. Therefore, we found no significant gene–noise interaction combinations after adjusting for covariates (*P* > 0.05), as shown in Table5. The cells intuitively reflect the gene–noise interaction see additional file 1 Figure S1.

**Table 5:** GMDR analysis for the best interaction combination models

Model	Cross-validation consistency	Testing balanced accuracy	*P*^a^
Noise	10/10	0.5527	0.0107
ACE, ACE2	6/10	0.5115	0.3770
ACE, ACE2, CYP	7/10	0.4817	0.9453
ACE, ACE2, CYP, noise	10/10	0.5084	0.3770

*P*^a^: *adjusted* BMI, eduation, hypertension family history, eat vegetable, physical exercise, age, length of service, TG, TC

## Discussion

The aetiology of EH is very complicated, and it is affected by a variety of genes, environmental factors, and their interactions. Its mechanism of action has not been ascertained so far.

Studies have found that the RAAS plays an important role in the regulation of blood pressure, the maintenance of water and salt balance, and the remodeling of cardiovascular tissues [15]. ACE is an important rate-limiting enzyme of RAAS. Agachan et al. [16] conducted a case–control study on 109 EH patients and 86 non-EH patients and found that the frequency of the D allele in the EH group was significantly higher than that in the control group. ACE2 can negatively regulate the RAAS system. Studies have shown that the ACE2 G8790A polymorphism is correlated with EH, and the risk of EH in people carrying the G allele is relatively high [8]. CYP11B2 is an important catalytic enzyme for the synthesis of aldosterone, which generates aldosterone by catalysing its production from deoxycorticosterone through a multistep reaction [17]. Studies have shown that both the C allele [18] and the T allele [19] are correlated with EH. In this study, the risk of having EH for ACE I/D DD genotype carriers was 1.99 times that for II genotype carriers (95% CI 1.14–3.51).

Research by Kohli et al. [20] showed that there is a significant interaction effect between genes, and the risk of EH from the interaction of the AGT and ACE genes is significant. Niu S et al. [21] conducted a case–control study on 52 patients with EH and 623 patients with normal blood pressure and found that there is a strong synergy between ACE I/D and CYP11B2-344T/C. AGT-6G, ACE I/D and CYP11B2 T-344C gene carriers have increased sensitivity to EH. Understanding gene–gene interactions can greatly help with the prevention and control of EH. In this study, the interaction between ACE2 G8790A and CYP11B2-344T/C based on the additive model showed that the two may have an interaction (*P* < 0.05). The interaction index S was 0.128, suggesting that the two may have a negative interaction. When a man has the AA genotype at ACE2 G8790A, also having the TC + CC genotype at CYP11B2-344T/C may reduce the risk of EH. The absolute value of AP was 0.856, suggesting that when men have the ACE2 G8790A AA genotype and the CYP11B2-344T/C TC + CC genotype, the rate of EH is affected by the interaction between the two is 85. The absolute value of RERI was 0.979, indicating that the effect of the interaction between the two on EH is 0.979 times that of other unknown factors. We found an ORINT of 0.485. In this study, other genes did not show any interaction based on the additive model.

Logistic regression was used to analyse the interaction between genes, and the results showed that ACE I/D and CYP11B2-344T/C polymorphisms may interact, based on the multiplicative model. The risk of having EH for ACE I/D DD and CYP11B2-344T/C TC genotype carriers was 3.04 times that for the ACE I/D II genotype and CYP11B2-344T/C TT genotype carriers (95% CI 1.25–7.39). The other genotypes did not show interactions in this study, based on the multiplicative model.

Occupational noise exposure is a harmful factor for workers. Yang et al. [22] conducted a meta–analysis that showed that workers affected by noise had a higher risk of EH than the control group. In this study, after adjusting for factors such as BMI, family history of hypertension, TG, and TC, it was found that noise exposure increased the risk of EH in male workers (OR = 1.52, 95% CI 1.04–2.22).

It is necessary to combine genetic factors with environmental factors to explore their impact on EH. Zawilla et al. [23] found that carriers of the ACE gene AG GG DD genotype were susceptible to hypertension under noise exposure. Hwang et al. [24] conducted a 21-year cohort study on 1301 aviation workers. The study found that workers with the AGT TT genotype had a higher risk of EH when exposed to occupational noise for a long time. In our analysis of the interaction between noise and ACE, ACE2 and CYP11B2 in both the additive and the multiplicative model, no noise–gene interaction were found.

When the multivariate logistic regression method was used, there was a problem of dimensionality. For example, a small sample size and a large number of independent variables may lead to an increase in type I errors. The GMDR method can adjust the covariates to improve the accuracy of prediction. Zhang et al. [25] studied the effect of gene–gene and gene–smoking interactions of CYP4A11 single-nucleotide polymorphisms on EH. Their results showed that there was a synergy between rs1126742 and rs3890011 and between rs1126742 and smoking according to the GMDR method. The GMDR method was used in this study and showed that there was no interaction between ACE, ACE2 and CYP11B2 and noise.

This study explored the influence of the interaction of ACE, ACE2, CYP11B2 gene polymorphisms and noise on EH. Some limitations of our research should be noted. First, the sample was not large, so the results of this study need to be verified in a study with a larger sample. Second, there were few gene loci typed in this study, and there are other genes related to EH in the RAAS system. In the future, more gene loci will be included to better study the relationships between hypertension and genetic and environmental factors. Finally, this study adopted a case–control research method, which may be affected by confounding factors. In the future, the scale of the research will be expanded to further study the influence of the interaction of genes and noise on EH.

## Conclusions

Noise exposure increases the risk of EH. The ACE DD genotype may be a susceptible genotype for essential hypertension. Carrying the DD genotype of ACE I/D and the TC genotype of CYP11B2-344T/C at the same time increases the risk of EH. The significance of this study is to screen high-risk groups and provide a theoretical basis for the prevention and treatment of EH.

## Supplementary Information

**Additional file 1: Table S1.** Primer Sequence of Each Gene Locus and PCR Reaction Conditions. **Table S2.** Primer Sequence of Each Gene Locus and PCR Reaction Conditions. **Table S3.** The Genotype and Allele Distributions of ACE, ACE2 and CYP11B2 Distribution in the EH Group and Control Group. **Table S4.** Analysis of the Multiplicative Interactions Between ACE, ACE2 and CYP11B2. **Table S5.** Analysis of Multiplication Interactions Between ACE, ACE2, CYP11B2 and Noise. **Figure S1.** The combined model of gene–noise interactions.

## Data Availability

The datasets generated and analyzed during the current study are not publicly available due other analyses are proceeding but are available from the corresponding author on reasonable request.

## Abbreviations

EH: Essential hypertension
RAAS: Renin–angiotensin–aldosterone system
JNC 7: The seventh US Joint National Committee on Detection, Evaluation and Treatment of Hypertension
BMI: Body mass index
TC: Total cholesterol
TG: Triglyceride
TWA: Time-weighted average
CNE: Cumulative noise exposure
WBGT: Wet bulb global temperature
RFLP: Restriction fragment length polymorphism
PCR: Polymerase chain reaction
HWE: Hardy–Weinberg equilibrium
OR: Odds ratios
CI: Confidence intervals
SPSS: Statistical Package for the Social Sciences
GMDR: Generalized multifactor dimensionality reduction
AP: Attribution proportion of the interaction
RERI: Relative excess risk of interaction
OR_(INT)_: Interaction odds ratio
NCBI: National Center for Biotechnology Information
MAF: Minimum allele frequency
SNP: Single Nucleotide Polymorphism
