# Supplementary material for: Effect of ACE, ACE2 and CYP11B2 gene polymorphisms and noise on essential hypertension among steelworkers in China: a case–control study
Source: BMC Med Genomics. 2022 Feb 8;15:22. doi: 10.1186/s12920-022-01177-0 (PMC8822663; doi:10.1186/s12920-022-01177-0)
Supplement: Supplementary file 1 — Additional file 1: Table S1. Primer Sequence of Each Gene Locus and PCR Reaction Conditions. Table S2. Primer Sequence of Each Gene Locus and PCR Reaction Conditions. Table S3. The Genotype and Allele Distributions of ACE, ACE2 and CYP11B2 Distribution in the EH Group and Control Group. Table S4. Analysis of the Multiplicative Interactions Between ACE, ACE2 and CYP11B2. Table S5. Analysis of Multiplication Interactions Between ACE, ACE2, CYP11B2 and Noise. Figure S1. The combined model of gene–noise interactions. [file 12920_2022_1177_MOESM1_ESM.docx]

**Effect of ACE, ACE2 and CYP11B2 Gene Polymorphisms and Noise on Essential Hypertension among steelworkers in China：a case-control study**

Xiaohong Zhang^1^, Ying Wang^2^, Yao Zheng^3^, Juxiang Yuan^1^, Junwang Tong^1^*, Jingya Xu^1^, Qinglin Li^1^, Peishuai Li^1^, Shoufang Jiang^1^, Zhaoyang Wang^4^, Feng Chai^1^, Xiangwen Li^1^

^1^ School of public health, North China University of Science and Technology.

^2^ Department of endocrinology, North China University of science and technology Affiliated Hospital.

^3^ Kailuan Occupational Disease Prevention Hospital.

^4^ Tangshan Hongci hospital.

Corresponding Author:

Junwang Tong,

School of Public Health, North China University of Science and Technology

No. 21 Bohai Avenue, Caofeidian Area, Tangshan, Hebei Province, P.R. China

Postal Code: 063210

Email: junwangtong@yeah.net

Phone: +8615932259005

**Additional file 1: Table S1.** Primer Sequence of Each Gene Locus and PCR Reaction Conditions

**Additional file 1: Table S2.** Primer Sequence of Each Gene Locus and PCR Reaction Conditions

**Additional file 1: Table S3.** The Genotype and Allele Distributions of ACE, ACE2 and CYP11B2 Distribution in the EH Group and Control Group

**Additional file 1: Table S4.** Analysis of the Multiplicative Interactions Between ACE, ACE2 and CYP11B2

**Additional file 1: Table S5.** Analysis of Multiplication Interactions Between ACE, ACE2, CYP11B2 and Noise

**Additional file 1: Figure S1.** The combined model of gene–noise interactions.

**Additional file 1: Table S1.** Primer Sequence of Each Gene Locus and PCR Reaction Conditions

| Gene | Locus | Primer sequence |
| --- | --- | --- |
| ACE | I/D | F (5’–CTGGAGACCACTCCCATCCTTTCT–3’) |
|  |  | R (5’–GATGTGGCCATCACATTCGTCAGAT–3’) |
| ACE2 | G8790A | F (5’–CATGTGGTCAAAAGGATATCT–3’) |
|  |  | R (5’–AAAGTAAGGTTGGCAGACAT–3’) |
| CYP | –344T/C | F (5’–GTTGACCACCAGGAGGAGAC–3’) |
|  |  | R (5’–GGAGAAGTCAGGTGCCTAAT–3’) |

**Additional file 1: Table S2.** Primer Sequence of Each Gene Locus and PCR Reaction Conditions

| Gene | Locus | pre–denaturation | denaturation | annealing | extension | Number of cycles | Terminal extension | Endonuclease |
| --- | --- | --- | --- | --- | --- | --- | --- | --- |
| ACE | I/D | 94℃ 5min | 94℃ 30S | 58℃ 40S | 72℃ 40S | 35 | 72℃ 5min |  |
| ACE2 | G8790A | 94℃ 5min | 94℃ 40S | 58℃ 30S | 72℃ 30S | 32 | 72℃ 5min | AluⅠ |
| CYP | –344T/C | 94℃ 5min | 94℃ 20S | 58℃ 30S | 72℃ 40S | 35 | 72℃ 5min | HaeⅢ |

**Additional file 1: Table S3.** The Genotype and Allele Distributions of ACE, ACE2 and CYP11B2 Distribution in the EH Group and Control Group

| Locus | Genotype | EH Group [n(%)] | Control Group[n(%)] | *χ2* | *P* |
| --- | --- | --- | --- | --- | --- |
| ACE(I/D) | II | 86(38.4) | 227(45.3) | 7.234 | **0.027** |
|  | ID | 101(45.1) | 224(44.7) |  |  |
|  | DD | 37(16.5) | 50(10.0) |  |  |
|  | I | 273(61.0) | 678(67.7) | 6.207 | **0.013** |
|  | D | 175(39.0) | 324(32.3) |  |  |
| ACE2(G8790A) | GG | 109(48.7) | 277(55.3) | 2.732 | 0.098 |
|  | AA | 115(51.3) | 224(44.7) |  |  |
|  | G | 218(48.7) | 554(55.3) | 5.464 | **0.019** |
|  | A | 230(51.3) | 448(44.7) |  |  |
| CYP11B2(–344T/C) | CC | 16(7.1) | 46(9.2) | 0.891 | 0.641 |
|  | TC | 86(38.4) | 193(38.5) |  |  |
|  | TT | 122(54.5) | 262(52.3) |  |  |
|  | C | 118(26.3) | 285(28.4) | 0.683 | 0.409 |
|  | T | 330(73.6) | 717(71.6) |  |  |

**Additional file 1: Table S4.** Analysis of the Multiplicative Interactions Between ACE, ACE2 and CYP11B2

| Gene–Gene | Genetype | | OR(95% *CI*) | OR^a^ (95% *CI*) |
| --- | --- | --- | --- | --- |
| ACE*CYP11B2 | II | TT | 1.00 | 1.00 |
|  | ID | TC | 1.06 (0.65–1.73) | 0.92 (0.53–1.60) |
|  | ID | CC | 0.84 (0.33–2.12) | 0.69 (0.24–1.97) |
|  | DD | TC | 2.46 (1.11–5.41) | **3.04 (1.25–7.39)** |
|  | DD | CC | 0.86 (0.16–4.58) | 0.84 (0.11–6.44) |
| *P* for interaction |  |  | 0.154 | **0.041** |
| ACE*ACE2 | II | GG | 1.00 | 1.00 |
|  | ID | AA | 1.75 (1.05–2.91) | 1.47 (0.81–2.65) |
|  | DD | AA | 2.07 (1.05–4.10) | 1.97 (0.90–4.28) |
| *P* for interaction |  |  | 0.202 | 0.253 |
| ACE2*CYP11B2 | GG | TT | 1.00 | 1.00 |
|  | AA | TC | 1.21 (0.75–1.94) | 1.11 (0.65–1.90) |
|  | AA | CC | 0.94 (0.43–2.05) | 0.88 (0.37–2.13) |
| *P* for interaction |  |  | 0.040 | 0.089 |

***Note.*** *OR^a^: adjusted* BMI, Eduation, Hypertension family history,Eat vegetable,Physical exercise,Age,Length of service,TG,

**Additional file 1: Table S5.** Analysis of Multiplication Interactions Between ACE, ACE2, CYP11B2 and Noise

| Gene–Noise | Genetype | Noise | OR(95%CI) | OR^a^(95%CI) |
| --- | --- | --- | --- | --- |
| ACE*Noise | II | <80 dB(A) | 1.00 | 1.00 |
|  | ID | ≥80 dB(A) | 1.64 (1.03–2.61) | 1.25 (0.56–2.77) |
|  | DD | ≥80 dB(A) | 3.21 (1.56–6.66) | 2.31 (0.84–6.36) |
| *P* for interaction |  |  | 0.932 | 0.590 |
| ACE2*Noise | GG | <80 dB(A) | 1.00 | 1.00 |
|  | AA | ≥80 dB(A) | 1.87 (1.20–2.90) | 1.41 (0.68–2.95) |
| *P* for interaction |  |  | 0.576 | 0.736 |
| CYP11B2*Noise | TT | <80 dB(A) | 1.00 | 1.00 |
|  | TC | ≥80 dB(A) | 1.47 (0.89–2.40) | 0.90 (0.42–1.92) |
|  | CC | ≥80 dB(A) | 0.95 (0.33–2.73) | 0.34 (0.09–1.25) |
| *P* for interaction |  |  | 0.362 | 0.198 |

***Note.*** *OR^a^: adjusted* BMI, Eduation, Hypertension family history, Eat vegetable, Physical exercise, Age, Length of service, TG, TC.


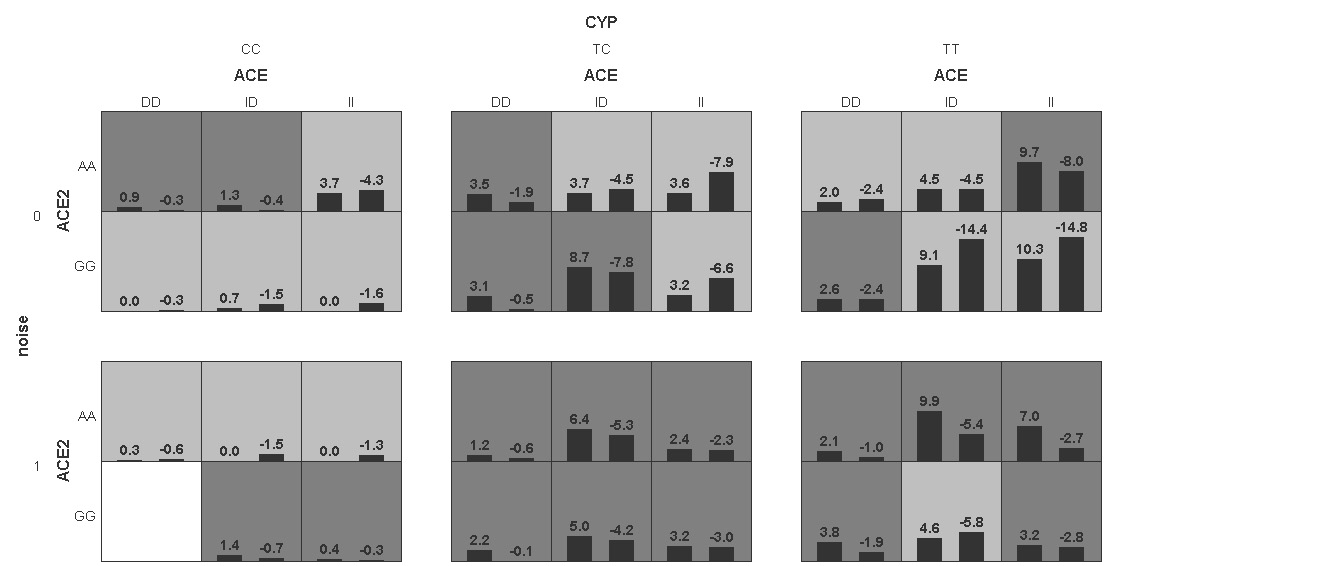


**Additional file 1: Figure S1.** The combined model of gene–noise interactions. The left side of the cell represents the case group, and the right side is the control group. High risks are represented by dark gray, low risks are represented by light gray, and blanks indicate that there is no effect or no such genotype in the population.
